# Capability of X-ray diffraction for the study of microstructure of metastable thin films

**DOI:** 10.1107/S2052252514021484

**Published:** 2014-10-28

**Authors:** David Rafaja, Christina Wüstefeld, Milan Dopita, Mykhaylo Motylenko, Carsten Baehtz

**Affiliations:** a Institute of Materials Science, TU Bergakademie Freiberg Germany; b Institute of Ion Beam Physics and Materials Research, Helmholtz Zentrum Dresden Rossendorf, Germany

**Keywords:** metastable thin films, microstructure, X-ray diffraction

## Abstract

Metastable phases are often used to design materials with outstanding properties, which cannot be achieved with thermodynamically stable compounds. In many cases, the metastable phases are employed as precursors for controlled formation of nanocomposites. This contribution shows how the microstructure of crystalline metastable phases and the formation of nanocomposites can be concluded from X-ray diffraction experiments by taking advantage of the high sensitivity of X-ray diffraction to macroscopic and microscopic lattice deformations and to the dependence of the lattice deformations on the crystallographic direction. The lattice deformations were determined from the positions and from the widths of the diffraction lines, the dependence of the lattice deformations on the crystallographic direction from the anisotropy of the line shift and the line broadening. As an example of the metastable system, the supersaturated solid solution of titanium nitride and aluminium nitride was investigated, which was prepared in the form of thin films by using cathodic arc evaporation of titanium and aluminium in a nitrogen atmosphere. The microstructure of the (Ti,Al)N samples under study was tailored by modifying the [Al]/[Ti] ratio in the thin films and the surface mobility of the deposited species.

## Abstract 2

The capability of X-ray diffraction for the microstructure investigations of metastable systems is illustrated on supersaturated and partially decomposed thin films of titanium aluminium nitrides with high aluminium content. The anisotropy of the elastic constants and their role in these investigations is discussed.

## 1. Introduction   

Thin films play an increasingly important role in contemporary life. They are used in electronics, optics, chemistry, as well as in energy and engineering applications. In the field of electronics, thin film technology is employed for the production of integrated circuits, memories, conductors, isolators and diffusion barriers. In the field of optics, thin films are utilized as reflection, antireflection and decorative coatings, for manufacturing of semiconductor lasers and for optical recording. The majority of magnetic and gas sensors, solar cells and batteries are nowadays also produced in the form of thin films. The engineering applications are dominated by wear and corrosion-resistant coatings, which typically improve the hardness and/or friction coefficient of the substrate materials.

Many applications exploit the outstanding properties of thin-film nanocomposites, which can be tailored to be superior in comparison with the properties of the individual constituents. Such nanocomposites consist of at least two nanoscaled phases, which are formed either during the deposition process or after the thin-film deposition. Numerous methods for the production of thin-film nanocomposites are based on the deposition of metastable compounds, which decompose in the thermodynamically stable phases either during the deposition process (Rafaja *et al.*, 2012 ▶) or during the subsequent annealing (Wüstefeld *et al.*, 2011 ▶; Haas *et al.*, 2013 ▶). The deposition of metastable compounds utilizes a limited mobility of the deposited atoms (adatoms), which can easily be manipulated through ion bombardment during the deposition (Sundgren *et al.*, 1983 ▶; Petrov *et al.*, 1989 ▶). In general, the microstructure and thus the physical properties of thins films can be reliably modified through the deposition parameters.

Although thin-film research was always supported by transmission electron microscopy, some microstructure features can be much better or more reliably determined using X-ray diffraction (XRD). The main advantage of XRD is its excellent accuracy regarding the interplanar spacing measurement, which predestines XRD for a precise quantification of the micromechanical behaviour of thin films. Furthermore, XRD offers statistically relevant microstructure information, as the XRD measurements are performed on relatively large sample volumes. In this contribution, the capability of XRD for the microstructure analysis of thin films is illustrated on the example of supersaturated and partially decomposed (Ti,Al)N solid solutions. The main topics of this study are the assessment of the stability of the supersaturated (Ti,Al)N, the estimation of the degree of its decomposition and the description of the effect of the partial phase segregation on the micromechanical properties of the thin films.

(Ti,Al)N thin films were first produced for electronic applications in 1970s (Wasa & Hayakawa, 1972 ▶), but a breakthrough for (Ti,Al)N thin films came with their application as oxidation-resistant, hard protective coatings (Knotek *et al.*, 1986 ▶; Münz, 1986 ▶). Since that time, (Ti,Al)N has been investigated as a model metastable system. The first attempt to describe the phase stability of (Ti,Al)N was made by Cremer *et al.* (1998 ▶), who reported that the metastable (Ti,Al)N with a cubic face-centered crystal structure of the NaCl-type exists up to approximately 65 mol% of AlN in TiN and remains metastable up to approximately 973 K. At higher temperatures, (Ti,Al)N decomposes into Ti-rich fcc-(Ti,Al)N and Al-rich w-(Al,Ti)N having a wurtzitic crystal structure. Later on, the stability of the metastable supersaturated (Ti,Al)N solid solution was assessed by using *ab initio* methods assuming that fcc-(Ti,Al)N decomposes spinodally into fcc-TiN and fcc-AlN (Mayrhofer *et al.*, 2006 ▶; Alling *et al.*, 2007 ▶). The spinodal decomposition was suggested to be followed by the transformation of fcc-AlN into its thermodynamically stable wurtzitic form (see *e.g.* Rachbauer *et al.*, 2011 ▶). Still, recent studies have shown that the spinodal decomposition followed by the w-AlN formation is not necessarily the sole mechanism of the fcc-(Ti,Al)N transformation. Alternatively, a direct formation of w-(Al,TiN) from fcc-(Ti,Al)N driven by the presence of stacking faults in fcc-(Ti,Al)N was considered as a competing process to the spinodal decomposition of fcc-(Ti,Al)N (Rafaja *et al.*, 2014 ▶).

In the present work, the phase composition, size and preferred local orientation of nanocrystallites and the micromechanical properties of supersaturated solid solutions with the overall chemical compositions Ti_0.53_Al_0.47_N and Ti_0.44_Al_0.56_N are correlated with the aluminium content and with the adatom mobility. The surface mobility of the adatoms was modified by the kinetic energy of the impinging ions, which were accelerated by the bias voltage applied during the cathodic arc evaporation (CAE) process. Furthermore, the effect of the internal lattice strain on the stabilization of the metastable fcc-(Ti,Al)N is discussed. The internal lattice strain affects the deformation energy which is, according to Cahn (1961 ▶), a factor influencing the Helmholtz free energy and thus the phase stability of metastable fcc-(Ti,Al)N. Finally, this study illustrates how the microstructure param­eters and the micromechanical characteristics determined using XRD can be employed to identify the particular decomposition mechanisms in metastable compounds.

## 2. Experimental details   

The (Ti,Al)N thin films under study were deposited in an industrial scale CAE facility of the Balzers RCS-type (Durand-Drouhin *et al.*, 2003 ▶). As substrates for the deposition, polished cemented carbide cutting inserts (SNUN-type, grade S40T) were used. The metallic atoms were sputtered from Ti–Al targets (PLANSEE Composite Materials GmbH), which were produced by a powder metallurgical route (Korb, 1988 ▶). Recently, it was shown that the CAE process activates the formation of intermetallic phases in powder metallurgically processed targets (Rafaja, Polzer *et al.*, 2011 ▶); thus these targets behave like the conventional cast targets. The chemical compositions of the targets were 50 at.% Ti:50 at.% Al and 40 at.% Ti:60 at.% Al, respectively. According to the results reported by Mayrhofer *et al.* (2006 ▶) and Alling *et al.* (2007 ▶), these aluminium concentrations were chosen to be within the spinodal decomposition region at the deposition temperature of approximately 723 K and below the AlN solubility in fcc-TiN (65.3 mol% of AlN in fcc-TiN) reported by Makino *et al.* (2005 ▶). Nitrogen was supplied directly from the deposition atmosphere, which was pure nitrogen with a working pressure of 3.2 Pa. The thickness of the thin films was about 2 µm.

Glow discharge optical emission spectroscopy (GDOES) performed on the deposited samples confirmed that the (Ti,Al)N films contained 50 at.% nitrogen as expected. The [Ti]/[Al] ratio was slightly higher in the thin films than in the targets. The energy of the ion impact on the surface of the deposited films, which along with the chemical composition was the main factor influencing the microstructure of the thin films, was adjusted by the bias voltage. The bias voltage (*U*_B_) was set to −40, −80 and −120 V in the respective deposition runs. In this range, the bias voltage has no effect on the chemical composition of the thin films measured using GDOES.

The X-ray diffraction experiments were performed in the glancing-angle diffraction mode (GAXRD) on the Rossendorf beamline (ROBL@BM20) at the European Synchrotron Radiation Facility (Grenoble). The measurements were carried out at a wavelength of 0.8857 Å. The data were collected using a scintillation detector, in front of which a Soller collimator was located. The angular range was 17–70° in 2θ, the step size 0.05° in 2θ. This angular range corresponds to the range of the diffraction vector 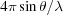 = 2.09–8.14 Å^−1^. The instrumental line broadening decreased nearly linearly with increasing diffraction angle from 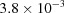
 Å^−1^ at 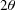 = 22° to 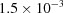
 Å^−1^ at 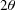 = 60°. The measuring time was 1.3 seconds per step.

The main reason for using GAXRD geometry for the thin-film experiments is to reduce the penetration depth of X-rays into the volume of the sample. When the penetration depth of the radiation is reduced, the diffraction signal from the thin film is more intense than the diffraction signal from the bulky substrate. In general, the penetration depth of X-rays is defined as the path of the radiation through the sample, which causes a decrease in the X-ray intensity to 1/*e* ≃ 37% of the primary intensity. Analogously in thin films measured using GAXRD geometry, the penetration depth of X-rays is defined as the distance of an infinitesimal slab of the material under study from the sample surface which delivers, after absorption, intensity that is equal to 1/*e* of the non-absorbed intensity. As the path of the X-rays in a thin film depends on the incident (γ) and outgoing angle (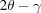), the penetration depth can be written as (see *e.g.* Rafaja *et al.*, 1997 ▶): 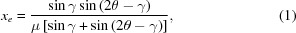
where 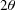 is the diffraction angle and μ is the mean linear absorption coefficient of the thin film. Other advantages of the GAXRD geometry are a weak dependence of the penetration depth on the diffraction angle and a concurrent strong decrease of the penetration depth with decreasing angle of incidence (Valvoda *et al.*, 1990 ▶).

In GAXRD experiments, it is reasonable to choose such an angle of incidence, for which the penetration depth is comparable with the film thickness, unless depth-resolved measurements are intended (Rafaja *et al.*, 1997 ▶). If the selected penetration depth is comparable with the film thickness, the information obtained from XRD is averaged over the whole thin film, while the signal from the substrate is sufficiently suppressed. In our experiments, the angle of incidence of the primary beam on the sample surface was 0.5°. This angle of incidence reduced the penetration depth of the radiation with a wavelength of 0.8857 Å below 1.5 µm as calculated according to equation (1) with the linear absorption coefficient of 59.5 cm^−1^. A smaller angle of incidence would further reduce the penetration depth, but the surface roughness of the samples would unnecessarily diminish the diffracted intensities (Suortti, 1972 ▶; Hermann & Ermrich, 1987 ▶).

The individual XRD lines from the measured diffraction patterns were fitted by the Pearson VII functions (Hall *et al.*, 1977 ▶) in order to obtain the line positions and the line broadening needed for further analyses. For selected samples, the diffraction patterns were subjected to Le Bail analysis (Le Bail *et al.*, 1988 ▶) and to Rietveld refinement (Rietveld, 1967 ▶, 1969 ▶) by using the *MAUD* routine (Ferrari *et al.*, 1996 ▶; Lutterotti *et al.*, 1999 ▶).

## 3. Results and discussion   

### 3.1. Effect of the chemical composition and mobility of the deposited atoms on the phase composition of metastable (Ti,Al)N thin films   

**Figure 1:**
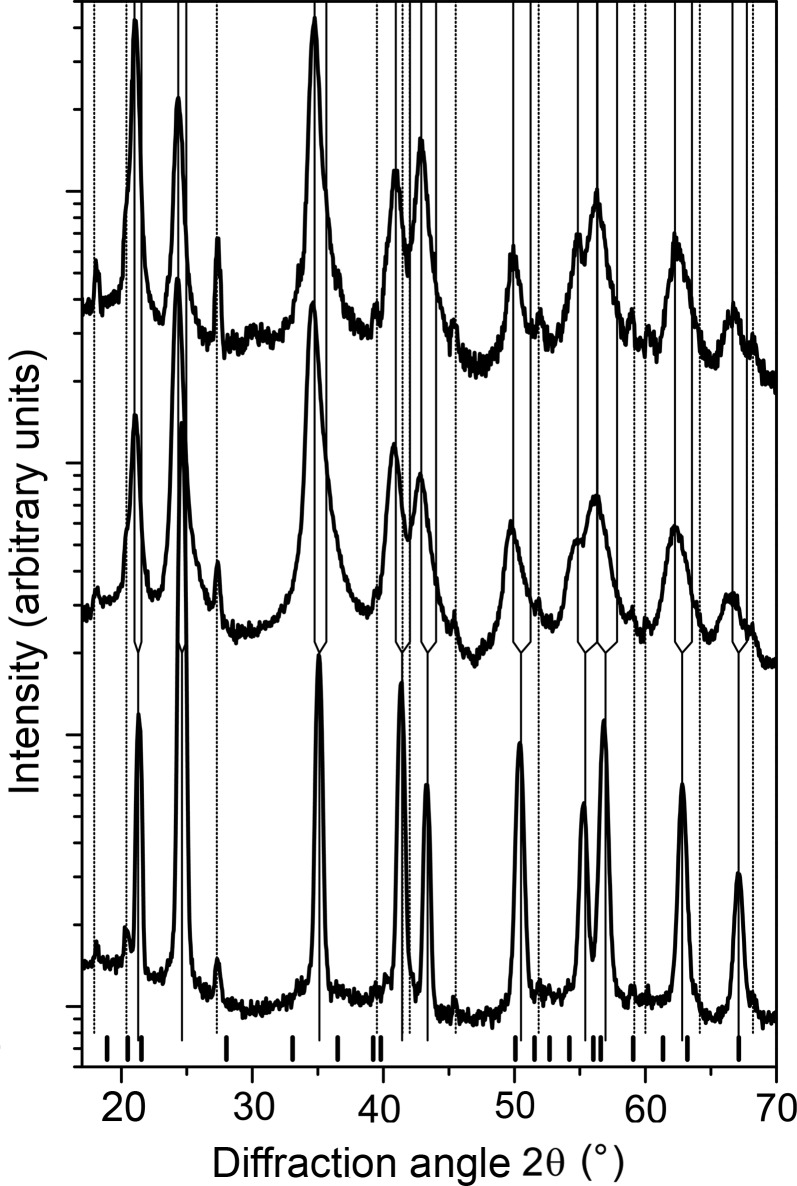
Diffraction patterns of samples with the overall chemical composition Ti_0.53_Al_0.47_N, which were deposited at *U*_B_ = −40 (bottom), −80 (middle) and −120 V (top). Vertical solid lines mark positions of the diffraction lines from the oversaturated fcc-(Ti,Al)N (in the lower part of the figure) and from Ti-rich and Al-rich fcc-(Ti,Al)N (in the upper part of the figure). The dotted lines mark positions of the diffraction lines from hex-WC, which is the main constituent of the substrate. The bars at the bottom of the figure show the positions of strong diffraction lines from w-(Al,Ti)N as calculated using the lattice parameters *a*_w_ = 3.11197 (2) and *c*_w_ = 4.98089 (4) Å from Paszkowicz *et al.* (2004 ▶).

**Figure 2:**
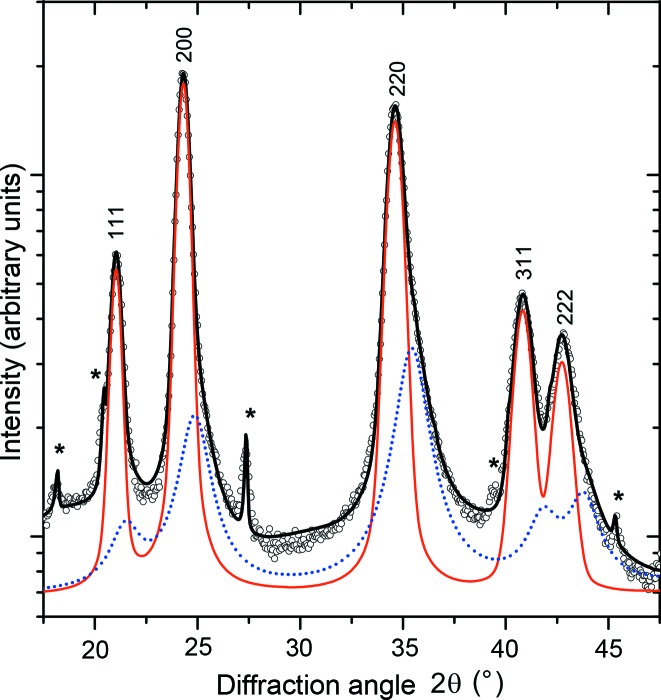
A part of the diffraction pattern of sample Ti_0.53_Al_0.47_N deposited at *U*_B_ = −80 V, which illustrates the decomposition of the oversaturated fcc phase into Ti-rich fcc-(Ti,Al)N and Al-rich fcc-(Al,Ti)N. The measured intensities are plotted as open circles and the total calculated intensities as a solid line. The intensities diffracted by the dominant phase in the fcc-(Ti,Al)N sample are plotted as a thin solid line (red) and those diffracted by fcc-(Al,Ti)N as a dotted line (blue). Diffraction indices of the fcc phases are displayed at the top of the figure. The asterisks mark the diffraction lines from hex-WC.

The XRD patterns of the samples with the overall chemical composition Ti_0.53_Al_0.47_N (Fig. 1 ▶) show mainly the diffraction lines from fcc-(Ti,Al)N and hexagonal tungsten carbide (hex-WC) which is the main constituent of the substrates. In samples deposited at *U*_B_ = −80 and −120 V, the presence of minor Al-rich fcc-(Al,Ti)N was indicated by weak diffraction maxima located at higher diffraction angles as compared with the positions of the XRD lines from the major phase, *i.e.* fcc-(Ti,Al)N. The positions and intensities of the diffraction lines of the respective fcc phase were obtained both from the fitting of the individual diffraction lines and from the Le Bail fit. The result of the Le Bail analysis is illustrated in Fig. 2 ▶ on sample Ti_0.53_Al_0.47_N deposited at *U*_B_ = −80 V. The thin solid (red) line shows the contribution of Ti-rich fcc-(Ti,Al)N to the diffraction pattern and the dotted (blue) line shows the contribution of Al-rich fcc-(Al,Ti)N. The superposition of the diffraction lines from Ti-rich fcc-(Ti,Al)N and Al-rich fcc-(Al,Ti)N causes an asymmetry in the XRD lines. The diffraction lines with even diffraction indices are more affected than the lines with odd diffraction indices (Wüstefeld *et al.*, 2010 ▶). The latter stay nearly symmetric, because the structure factors of the diffraction lines from fcc-(Al,Ti)N with odd diffraction indices are low (Rafaja *et al.*, 2014 ▶). Distinct XRD lines from w-(Al,Ti)N were not observed. Instead, maxima of the diffuse scattering appeared near the diffraction angles which correspond to the crystalline w-(Al,Ti)N. These line positions are labelled by short bars located at the bottom of Fig. 1 ▶.

Fig. 1 ▶ also illustrates a larger XRD line broadening in the samples deposited at *U*_B_ = −80 and −120 V in comparison with the line broadening observed in the sample deposited at *U*_B_ = −40 V. The maximum of the XRD line broadening is reached in the sample deposited at *U*_B_ = −80 V. In general, a higher bias voltage makes the ion impact more intense. A higher energy for the ion impact diminishes the mean free path of the deposited atoms on the surface and thus the adatom mobility, but it simultaneously heats up the sample surface that increases the surface diffusivity of the adatoms (Oura *et al.*, 2003 ▶) at the highest bias voltages. This interference of the involved phenomena has already been discussed by Wüstefeld *et al.* (2010 ▶), who showed that the minimum for the surface mobility occurs at *U*_B_ = −80 V in this particular deposition system.

**Figure 3:**
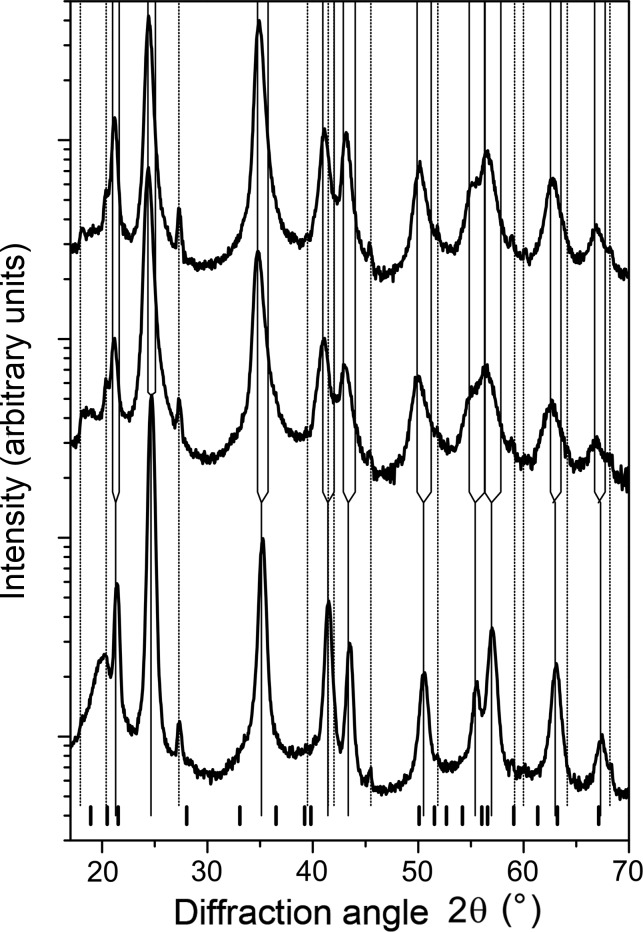
Diffraction patterns of samples with the overall chemical composition Ti_0.44_Al_0.56_N, which were deposited at *U*_B_ = −40 (bottom), −80 (middle) and −120 V (top). The meaning of the vertical lines and the bars is the same as in Fig. 1 ▶.

**Figure 4:**
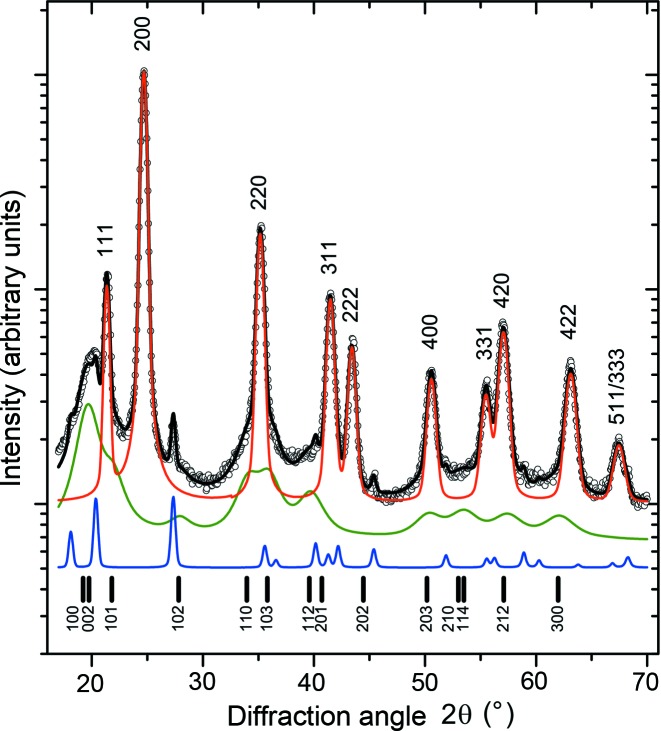
Le Bail fit of the diffraction pattern of sample Ti_0.44_Al_0.56_N deposited at *U*_B_ = −40 V. The measured intensities are plotted as open circles, the calculated intensities as solid lines. The contributions of individual phases to the total calculated intensity are mutually shifted for clarity. The intensities diffracted by fcc-(Ti,Al)N (red line) are at the top, the intensities from w-(Al,Ti)N (green line) in the middle and the intensities from hex-WC (blue line) at the bottom. The upper diffraction indices indicate the diffraction lines from fcc-(Ti,Al)N while the indices at the bottom of the figure indicate the diffraction lines from w-(Al,Ti)N.

In samples with the overall chemical composition Ti_0.44_Al_0.56_N (Fig. 3 ▶), the XRD lines from fcc-(Ti,Al)N and hex-WC are complemented by broad diffraction maxima from w-(Al,Ti)N. The most pronounced diffraction maximum from w-(Al,Ti)N can be found at around 20° in 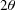. In samples deposited at *U*_B_ = −80 and −120 V, a similar asymmetry of the fcc diffraction lines with even diffraction indices was observed as in the samples of Ti_0.53_Al_0.47_N. The simulation of the diffraction pattern for the wurtzitic structure and a subsequent Rietveld refinement using the Le Bail fit (Le Bail *et al.*, 1988 ▶) in *MAUD* (Ferrari *et al.*, 1996 ▶; Lutterotti *et al.*, 1999 ▶) performed for sample Ti_0.44_Al_0.56_N deposited at *U*_B_ = −40 V revealed that the strongest (and narrowest) diffraction lines from w-(Al,Ti)N are 100, 002, 110, 112, 210, 114 and 300 (*cf*. Fig. 4 ▶). These diffraction indices fulfil the relationship *h* = *k* or *l* = 0. According to Warren (1969 ▶), the diffraction lines with these diffraction indices are not broadened by the stacking faults, which are randomly distributed on the hexagonal lattice planes 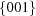. The apparently strong diffraction lines 103, 203 and 212 imitate the diffuse scattering from the fcc structure.

The formation of stacking faults in w-(Al,Ti)N accompanies a direct transition between fcc-(Ti,Al)N and w-(Al,Ti)N as reported by Rafaja *et al.* (2014 ▶). This transformation mechanism, which is facilitated by the stacking faults located on the wurtzitic lattice planes 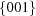 and on the fcc lattice planes 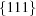, requires the orientation relationship 
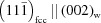
 and 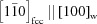 (or symmetrically equivalent ones) between the cubic and the wurtzitic counterparts. These orientation relationships were previously found in similar (Ti,Al)N thin films by using local fast Fourier transformation of high-resolution transmission electron micrographs (Rafaja, Wüstefeld *et al.*, 2011 ▶). Analogue transformation pathways for the phase transitions in AlN were reported recently by Schmerler & Kortus (2014 ▶).

Furthermore, the Rietveld refinement of the diffraction pattern measured in sample Ti_0.44_Al_0.56_N deposited at *U*_B_ = −40 V revealed the lattice parameters of w-(Al,Ti)N *a*_w_ = 3.033 (5) and *c*_w_ = 5.16 (1) Å. In comparison with the intrinsic lattice parameters of w-AlN, *a*_w_ = 3.11197 (2) and *c*_w_ = 4.98089 (4) Å (Paszkowicz *et al.*, 2004 ▶), the lattice parameter *a*_w_ measured in this thin film is smaller, whereas *c*_w_ is larger. A similar distortion of the elementary cell was reported by Christensen & Gorczyca (1994 ▶) for w-AlN under hydrostatic pressure, who gave *a*_w_ = 3.081 and *c*_w_ = 5.031 Å. In our case, the reduction of *a*_w_ is caused by the intergrowth of the cubic and wurtzitic regions with the mutual orientation 
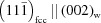
 and 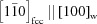 and with the habitus planes 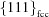 and 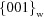. This kind of heteroepitaxy implies a convergence of the interatomic distances within the habitus plane of the fcc and wurtzitic structures, which are equal to *a*_fcc_(2)^−1/2^ and *a*_w_ for the respective crystal structure as calculated from the distances between the metallic atoms (Rafaja *et al.*, 2014 ▶). As *a*_fcc_(2)^−1/2^ of (Ti,Al)N is always less than *a*_w_ [even for Al-free fcc-TiN, where *a*_fcc_ = 4.242 Å and *a*_fcc_(2)^−1/2^ = 3.000 Å], the elementary cell of w-AlN is compressed in the 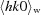 directions and expands in the non-constrained [001]_w_ direction.

Another consequence of such intergrowth of the cubic and wurtzitic domains is the relatively high observed intensity of the diffraction line 002 from w-(Al,Ti)N, *cf*. Figs. 3 ▶ and 4 ▶. The high intensity of this diffraction line stems from the transient regions, in which the local crystal structure alternates between fcc-(Ti,Al)N and w-(Al,Ti)N and which consequently contain a high density of the stacking faults on the lattice planes 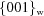 and 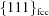. In such regions, the translation periodicity is almost broken with the exception of the 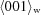 and 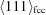 directions, in which the heteroepitaxially grown cubic and wurtzitic domains are mutually coherent and produce an enhanced coherently diffracted intensity.

The XRD line broadening in the samples with the chemical composition Ti_0.44_Al_0.56_N behaves similarly to the XRD line broadening observed in samples with the chemical composition Ti_0.53_Al_0.47_N. The maximum line broadening was measured in the samples deposited at the bias voltage of −80 V. Even at *U*_B_ = −40 V, the XRD lines are broader in sample Ti_0.44_Al_0.56_N than in sample Ti_0.53_Al_0.47_N.

### 3.2. Micromechanical properties of metastable (Ti,Al)N thin films   

Additional information about the micromechanics of metastable (Ti,Al)N thin films were obtained from the lattice parameters of fcc-(Ti,Al)N. Within the Reuss model (Reuss, 1929 ▶), the cubic lattice parameters measured in a thin film being under equi-axial macroscopic stress (σ) depend both on the inclination of the diffraction vector from the normal direction, ψ, and on the crystallographic direction *hkl* (see *e.g.* Rafaja *et al.*, 2010 ▶)
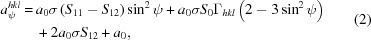
where 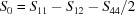; 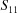, 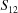 and 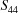 are the three independent elements of the cubic compliance tensor and 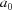 is the stress-free lattice parameter of a cubic material. The dependence of the lattice parameters on the crystallographic direction follows the cubic invariant:
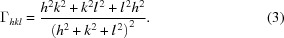
In GAXRD geometry, ψ is related to the diffraction angle 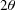 and to the angle of incidence of the primary beam on the sample surface (γ):
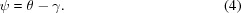
It should be noted that although equation (2) is based on the Reuss approximation, an analogous dependence of the measured lattice parameters on the crystallographic direction can be obtained for the Eshelby–Kröner model (Eshelby, 1957 ▶; Kröner, 1958 ▶) and for the Vook & Witt (1965 ▶) model, because all three models reveal the same functional dependence of the X-ray elastic constants on the cubic invariant 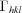 [see *e.g.* Welzel *et al.* (2005 ▶)].

**Figure 5:**
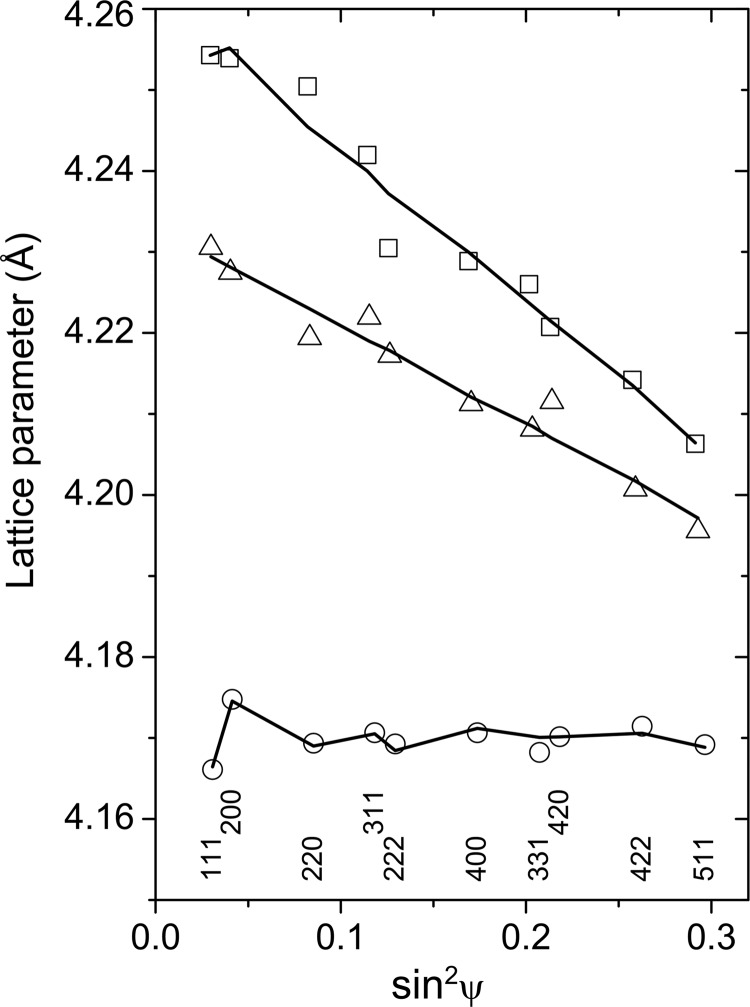
Dependence of the lattice parameters on 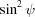 and on the diffraction indices as measured in samples with chemical composition Ti_0.53_Al_0.47_N, which were deposited at *U*_B_ = −40 (circles), −80 (boxes) and −120 V (triangles). The solid lines represent the least-squares fit of the measured lattice parameters according to equation (2).

**Figure 6:**
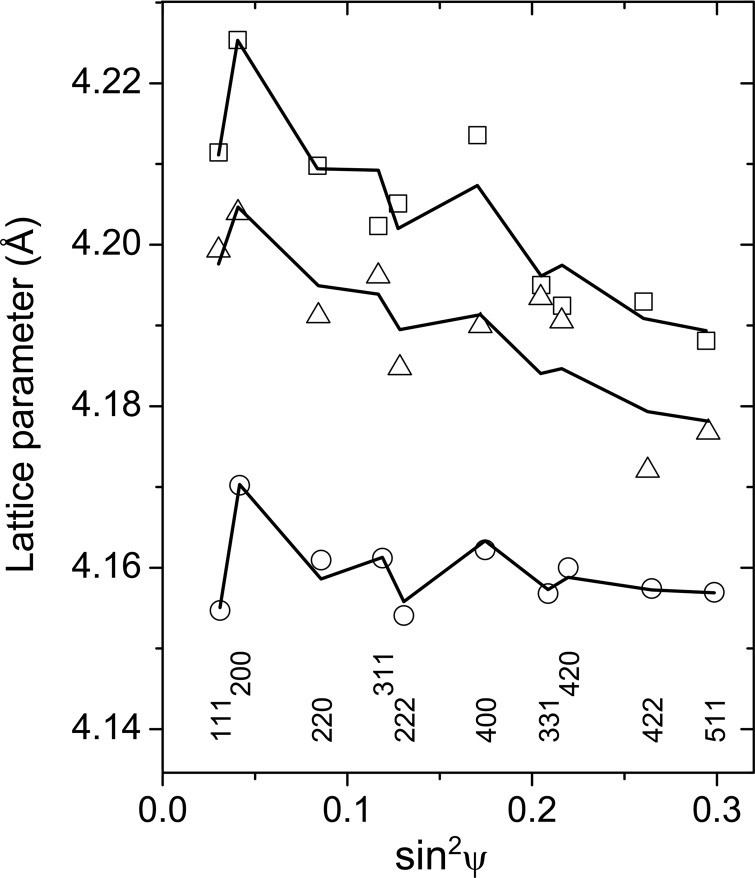
Dependence of the lattice parameters on 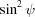 and on the diffraction indices as measured for samples Ti_0.44_Al_0.56_N deposited at *U*_B_ = −40 V (circles), −80 V (boxes) and −120 V (triangles). The solid lines represent the least-squares fit of the measured lattice parameters according to equation (2).

**Figure 7:**
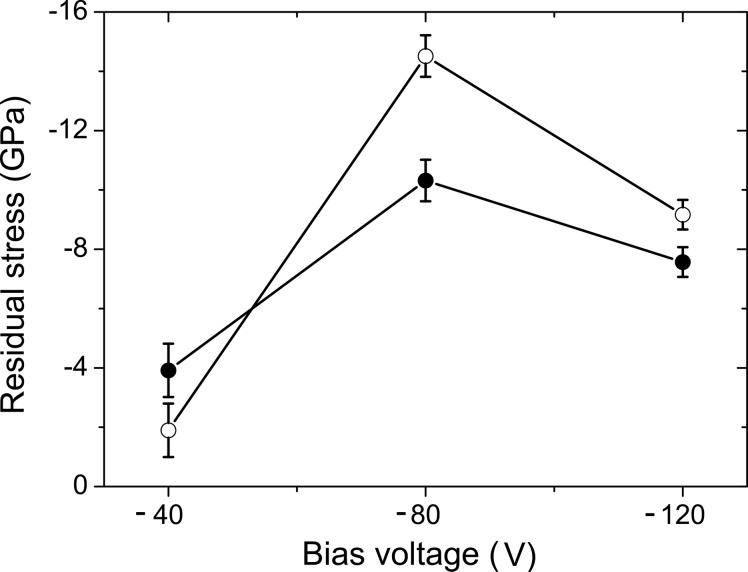
Dependence of the residual stress on the bias voltage for samples with chemical compositions Ti_0.53_Al_0.47_N (open symbols) and Ti_0.44_Al_0.56_N (filled symbols). The residual stress was calculated using the Reuss approach with the elastic constants from Tasnádi *et al.* (2010 ▶).

The results of least-squares fits for samples Ti_0.53_Al_0.47_N (Fig. 5 ▶) and Ti_0.44_Al_0.56_N (Fig. 6 ▶) show that the best agreement between the model from equation (2) and the experimental data can be achieved for samples deposited at *U*_B_ = −40 V, although the sample Ti_0.44_Al_0.56_N produces a large amount of the diffuse scattering (*cf*. Figs. 1 ▶ and 3 ▶), which was explained above by the presence of segregated wurtzitic regions (Fig. 4 ▶). According to equation (2), the slopes of the 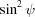 plots (Figs. 5 ▶ and 6 ▶) are proportional to the residual stress σ. The residual stresses in all samples under study are summarized in Fig. 7 ▶ as calculated from the parameters 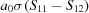 and 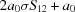, which were obtained from the least-squares fit of equation (3) to the measured lattice parameters, and from the elastic constants *S*_11_ = 2.59 TPa^−1^ and *S*_12_ = −0.637 TPa^−1^ calculated by Tasnádi *et al.* (2010 ▶). It is worth noting that in samples with high residual stress (deposited at *U*_B_ = −80 and −120 V), the Al-rich fcc-(Al,Ti)N was found (*cf*. Fig. 2 ▶). As fcc-AlN is a high-pressure phase (Vollstädt *et al.*, 1990 ▶), the Al-rich fcc-(Al,Ti)N is considered to be stabilized by the residual stress present in the samples deposited at high bias voltages.

**Figure 8:**
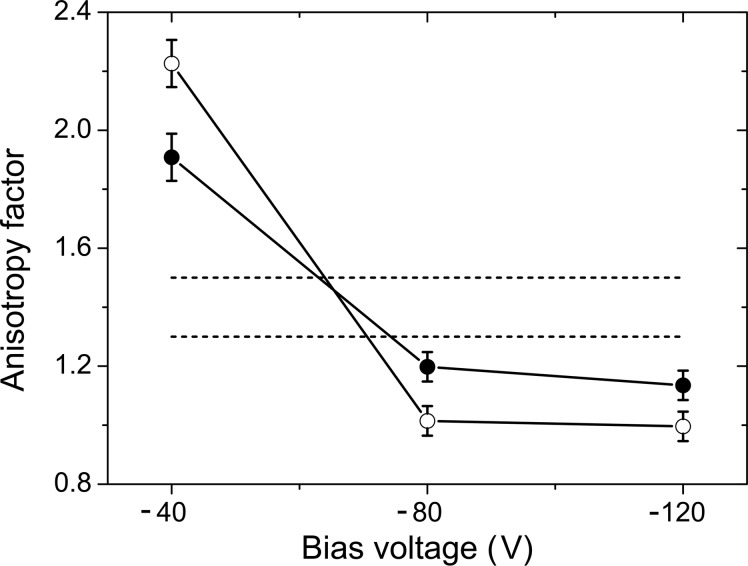
Anisotropy factors calculated using equation (6) for fcc-(Ti,Al)N in samples Ti_0.53_Al_0.47_N (open symbols) and Ti_0.44_Al_0.56_N (filled symbols). The horizontal dashed lines are the anisotropy factors reported by Tasnádi *et al.* (2010 ▶) for the respective compound. The smaller theoretical anisotropy factor corresponds to Ti_0.53_Al_0.47_N, the higher one to Ti_0.44_Al_0.56_N.

From the refined factors 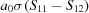 and 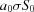, the anisotropy of the cubic elastic constants (see *e.g.* Tasnádi *et al.*, 2010 ▶),
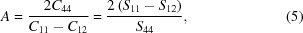
can be calculated according to
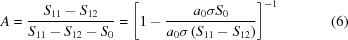
because 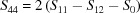. The anisotropy factors calculated by using equation (6) are summarized in Fig. 8 ▶ and compared with the anisotropy factors, which were determined by Tasnádi *et al.* (2010 ▶) from the *ab initio* calculations performed on the titanium aluminium nitrides with corresponding chemical compositions.

The anisotropy factors determined for both samples deposited at *U*_B_ = −40 V are much higher than the anisotropy factors predicted from the *ab initio* calculation. High-anisotropy factors obtained from the XRD experiment are a consequence of a large difference between the measured lattice parameters 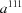 and 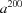 (Figs. 5 ▶ and 6 ▶) in samples that are under relatively low residual compressive stress (Fig. 7 ▶). As the difference between 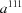 and 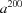 depends on σ and 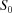 through the factor 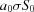 in equation (2), the elastic constant 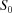 must be increased in order to describe the large difference between 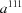 and 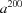 at low residual stresses. Finally, the increase of 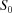 leads to an apparent increase of the anisotropy factor calculated according to equation (6).

In the decomposed (Ti,Al)N thin films, the anisotropy of the elastic constants and especially the difference between 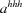 and 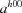 are intensified by the interaction between fcc-(Ti,Al)N and w-(Al,Ti)N grown heteroepitaxially with the habitus planes 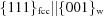 as already discussed in §3.1. As the distances between the metallic atoms within the 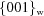 planes of w-(Al,Ti)N are larger than the interatomic distances within the 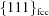 planes of fcc-(Ti,Al)N, w-(Al,Ti)N is compressed mainly in the 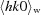 directions and expanded mainly in the perpendicular direction [001]_w_. The elementary cell of fcc-(Ti,Al)N is expanded mainly in the directions perpendicular to 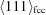, *e.g.* 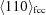 and 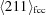, and compressed in one of the equivalent crystallographic directions 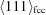, which is perpendicular to the respective habitus plane. In analogy with equation (2), the deformation of the fcc lattice caused by the heteroepitaxy with w-(Al,Ti)N can be described as a linear function of 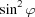, where ϕ is the angle between the cubic lattice planes 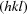 and 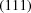:
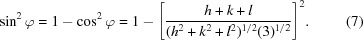
In samples with intergrown fcc-(Ti,Al)N and w-(Al,Ti)N, the interplanar distances of the lattice planes with 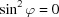 such as (111) or (222) shrink, whereas the interplanar distances of the lattice planes with 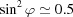, such as (200), (400), (420) or (511), are almost unaffected. Such a dependence of 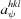 on 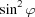 correlates with the 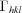 dependence of 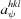 from equation (2), which apparently enhances the elastic anisotropy of the nanocomposite, in particular if the anisotropy is calculated from equation (6) using the parameters 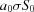 and 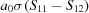 obtained from fitting the experimental data by using equation (2).

On the contrary, the anisotropy factors of samples deposited at *U*_B_ = −80 and −120 V are lower than expected (Fig. 8 ▶). In these samples, the low value of 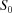 can be interpreted as an effective increase to the shear component of the compliance, 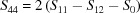, or as an effective reduction of the shear stiffness 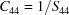, which facilitates both the formation of the stacking faults in fcc-(Ti,Al)N (Rafaja *et al.*, 2014 ▶) and the shear-induced phase transitions in AlN (Schmerler & Kortus, 2014 ▶). However, strongly faulted w-(Al,Ti)N does not significantly contribute to the coherently scattered X-rays, thus the diffraction maxima from w-(Al,Ti)N become weaker, as it is clearly visible in Fig. 3 ▶.

**Figure 9:**
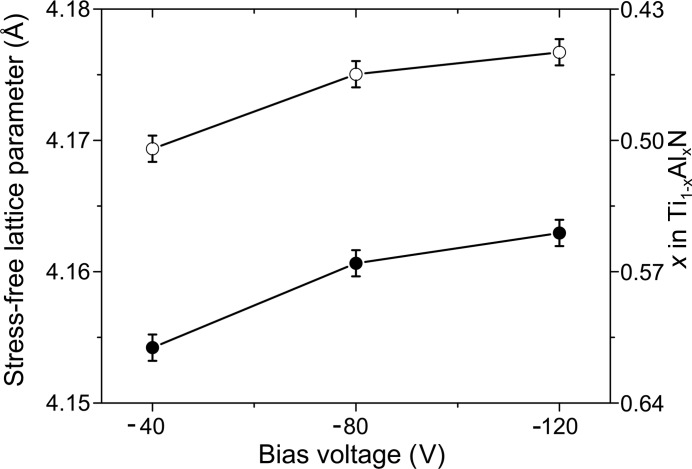
Stress-free lattice parameters of fcc-(Ti,Al)N in samples Ti_0.53_Al_0.47_N (open symbols) and Ti_0.44_Al_0.56_N (filled symbols). The stress-free lattice parameters were converted to the aluminium content in Ti_1−*x*_Al_*x*_N using the Vegard-like dependence *a*_0_ = 4.2418 − 0.1432*x* (Å) from Rafaja, Wüstefeld *et al.* (2008 ▶).

To some extent, the low-anisotropy factors determined for samples deposited at *U*_B_ = −80 and −120 V are caused by the segregation of AlN from fcc-(Ti,Al)N. According to Tasnádi *et al.* (2010 ▶), the elastic anisotropy factor of Ti_1−*x*_Al_*x*_N decreases with decreasing *x*, until it reaches unity (as in an isotropic medium) for *x* ≃ 0.27. Using the XRD data, the Al content in fcc-(Ti,Al)N can be estimated from the Vegard-like dependence of the stress-free lattice parameter of Ti_1−*x*_Al_*x*_N on *x* (Rafaja, Wüstefeld *et al.*, 2008 ▶), *a* = 4.2418 − 0.1432*x*. The stress-free lattice parameters of all samples under study and the corresponding Al concentrations are summarized in Fig. 9 ▶. The stress-free lattice parameters were determined from the refined factor 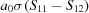 by using the residual stress and the elastic constants 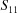 and 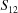 discussed above. As already indicated by the shift of the measured lattice parameters in Figs. 5 ▶ and 6 ▶, the stress-free lattice parameter increases with increasing bias voltage for both chemical compositions, *i.e.* for Ti_0.53_Al_0.47_N (Fig. 5 ▶) and Ti_0.44_Al_0.56_N (Fig. 6 ▶). Still, the amount of Al in fcc-(Ti,Al)N ranges only between *x* = 0.50 and 0.45 in Ti_0.53_Al_0.47_N and between *x* = 0.60 and 0.55 in Ti_0.44_Al_0.56_N (Fig. 9 ▶). These composition ranges correspond to the variations of the anisotropy factors between 1.25 and 1.33 for Ti_0.53_Al_0.47_N and between 1.42 and 1.50 for Ti_0.44_Al_0.56_N (Tasnádi *et al.*, 2010 ▶), which cannot explain the observed anisotropy factors from Fig. 8 ▶. Therefore, the main reason for the observed changes in the elastic anisotropy is the interaction between the fcc-(Ti,Al)N crystallites and their Al-enriched vicinity.

### 3.3. Fragmentation of the microstructure   

**Figure 10:**
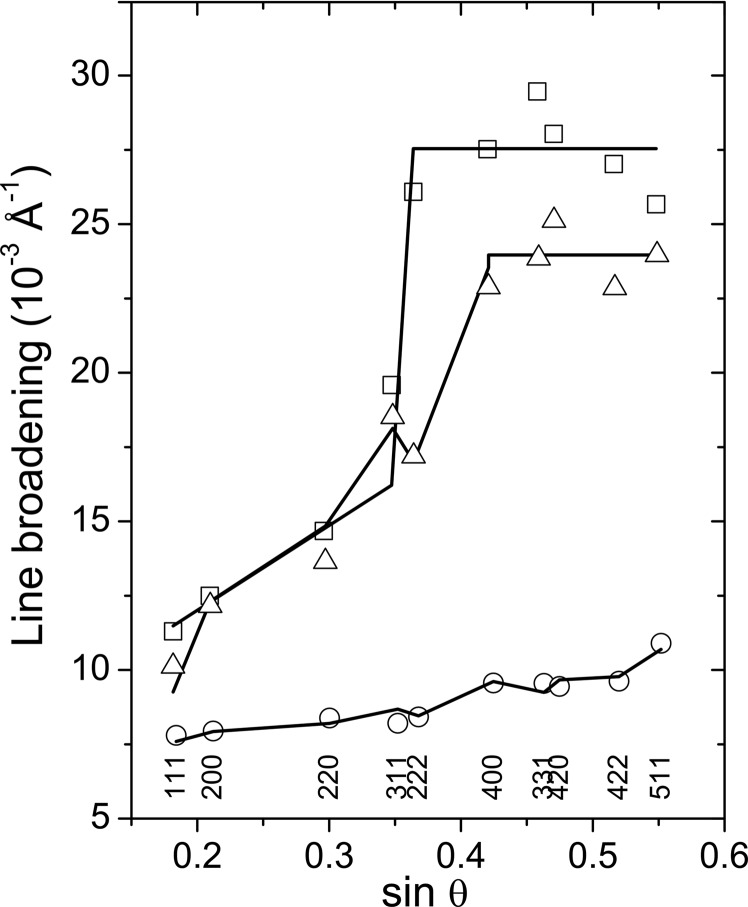
Diffraction line broadening measured for fcc-(Ti,Al)N in Ti_0.53_Al_0.47_N and plotted as a function of 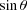. Diffraction indices are indicated at the bottom of the figure. The line broadening in the sample deposited at *U*_B_ = −40 V (circles) was approximated by equation (8). The same approximation was used for the line broadening measured at low diffraction angles in the samples deposited at *U*_B_ = −80 (boxes) and −120 V (triangles). The line broadening measured in these samples at high diffraction angles was assumed to be constant.

Complementarily, the segregation of AlN from fcc-(Ti,Al)N can be concluded from the XRD line broadening, which is shown in the form of the Williamson & Hall (1953 ▶) plot, *i.e.* as a function of 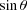, in Figs. 10 ▶ and 11 ▶ for Ti_0.53_Al_0.47_N and Ti_0.44_Al_0.56_N, respectively. The line width (FWHM) measured for samples deposited at *U*_B_ = −40 V (open circles in Figs. 10 ▶ and 11 ▶) complies with the modified Williamson–Hall dependence,
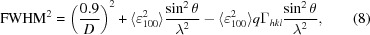
which was suggested by Ungár *et al.* (1999 ▶) to describe the effect of dislocations on the XRD line broadening in cubic materials. An important statement of equation (8) is that the line broadening depends on the cubic invariant 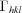 from equation (3), which represents the dependence of the contrast factors of dislocations and the dependence of the local variance of the interplanar spacings 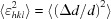 on the crystallographic direction *hkl* (Ungár *et al.*, 1999 ▶). The meaning of other symbols in equation (8) is as follows: *D* is the mean crystallite size, θ is a half of the diffraction angle, λ is the wavelength of the X-ray radiation and *q* is the amount of the crystallographic anisotropy.

The least-squares fitting of equation (8) to the FWHMs measured in samples deposited at *U*_B_ = −40 V yielded *D* = 12.4 (8) nm, 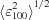 = 23 (2) × 10^−3^ and *q* = 1.1 (1) for Ti_0.53_Al_0.47_N, and *D* = 10.3 (9) nm, 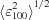 = 42 (2) × 10^−3^ and *q* = 1.2 (1) for Ti_0.44_Al_0.56_N. According to Ungár *et al.* (1999 ▶), the anisotropy factor in the contrast factor of dislocations [*q* from equation (8)] depends on the ratio of the elastic constants *C*_12_ and *C*_44_ and on the elastic anisotropy *A*. For an fcc structure with *C*_12_/*C*_44_ = 0.75 and *A* = 1.3–1.5 (Tasnádi *et al.*, 2010 ▶), the anisotropy factors of screw and edge dislocations vary between 1.43 and 1.66 and between 0.43 and 0.66, respectively, as calculated using the parametric formula given by Ungár *et al.* (1999 ▶).

Although the anisotropy factors determined from the XRD line broadening (Figs. 10 ▶ and 11 ▶) come within the limits expected for dislocations, the observed anisotropy of the XRD line broadening is not necessarily caused solely by dislocations in the metastable (Ti,Al)N thin films. Rather, it is related to the anisotropic response of the cubic structure in the vicinity of the microstructure defects, which result from the segregation of AlN from the supersaturated fcc-(Ti,Al)N solid solution. Correspondingly, the increase of the line broadening with increasing 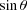, which corresponds to a higher microstrain 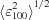, is more pronounced in sample Ti_0.44_Al_0.56_N with a higher Al content, because it is more susceptible to decomposition than the sample Ti_0.53_Al_0.47_N.

Development of such microstrain was discussed by Wüstefeld *et al.* (2011 ▶), who explained the microstrain by local concentration fluctuations of Ti and Al in (Ti,Al)N. The local concentration fluctuations in as-deposited metastable (Ti,Al)N coatings were detected formerly through atom-probe analysis (Rachbauer *et al.*, 2009 ▶). It follows from the crystallographic anisotropy of the line broadening observed in (Ti,Al)N samples deposited at *U*_B_ = −40 V that the response of the cubic structure to the local lattice strains caused by the concentration fluctuations is analogous to the anisotropic lattice deformation near dislocations; assumed by Klimanek & Kužel (1988 ▶) to be the reason for the dependence of the contrast factors of dislocations on the crystallographic direction.

In the samples deposited at higher bias voltages (*U*_B_ = −80 and −120 V) and thus at a lower adatom mobility, the line broadening (boxes and triangles in Figs. 10 ▶ and 11 ▶) is significantly higher than the line broadening in the samples deposited at higher mobility of the deposited species (*U*_B_ = −40 V). Furthermore, the pronounced dependence of the line width on the crystallographic direction and even the linear dependence of the line broadening on 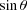 disappear. At high diffraction angles, the line broadening increases abruptly and remains nearly constant.

Originally, this phenomenon was explained by a partial overlap of strongly broadened reciprocal lattice points belonging to slightly mutually misoriented neighbouring nanocrystallites (Rafaja *et al.*, 2004 ▶). The extent of the overlap depends on the magnitude of the diffraction vector, *i.e.* on the distance of the respective reciprocal lattice point from the origin of reciprocal space, and on the mutual misorientation of the crystallites. Near the origin of the reciprocal space, *i.e.* at small diffraction vectors, the strongly broadened reciprocal lattice points overlap, which is recognized by XRD as a high partial coherence of the neighbouring nanocrystallites. Consequently, the resulting XRD lines become narrower in this region, which can be interpreted alternatively that XRD cannot distinguish the adjacent nanocrystallites with nearly identical orientations from each other. Instead, the Williamson–Hall analysis identifies agglomerates of such nanocrystallites (nanocrystalline clusters) as large but highly defective crystallites. The corresponding defects are mainly represented by ‘small angle’ boundaries between the mutually misoriented crystallites (Rafaja, Klemm *et al.*, 2008 ▶).

For partially coherent crystallites, the slope of the Williamson–Hall plot is basically determined by the loss of the partial coherence with increasing magnitude of the diffraction vector. Far from the origin of the reciprocal space, *i.e.* at large diffraction vectors, the partial coherence of neighbouring nanocrystallites disappears. In this region, XRD recognizes individual nanocrystallites as coherently diffracting domains and their intrinsic defects as the only source of the microstrain. Consequently, the XRD line broadening is much higher than in the vicinity of the origin of the reciprocal space and nearly constant, because the concentration of intrinsic defects (*e.g.* dislocations) in nanocrystallites is low.

In metastable thin films, the loss of the partial coherence of nanocrystallites can be used as an indicator of the fragmentation of the thin-film microstructure (Rafaja, Wüstefeld *et al.*, 2008 ▶). For the Ti_0.53_Al_0.47_N sample, the analysis of the cluster size in terms of equation (8), *i.e.* from the intercept of the line broadening measured at low diffraction angles with the ordinate, revealed 10 (2) nm for both bias voltages (−80 and −120 V). The size of individual crystallites was 3.6 (4) nm for *U*_B_ = −80 V and 4.2 (5) nm for *U*_B_ = −120 V. In the Ti_0.44_Al_0.56_N sample, the size of the fcc-(Ti,Al)N clusters was 12 (1) and 16 (2) nm for *U*_B_ = −80 and −120 V, respectively. The respective crystallite sizes were 3.2 (4) and 4.2 (4) nm.

### 3.4. Microstructure of supersaturated (Ti,Al)N solid solution   

Based on the results above, the microstructure of the supersaturated (Ti,Al)N solid solutions and the underlying mechanisms of the microstructure formation can be summarized as follows. The supersaturated (Ti,Al)N solid solutions are metastable compounds, which tend to decompose into the thermodynamically stable fcc-TiN and w-AlN. The chemical driving force for the decomposition increases with increasing aluminium content. The stabilization of metastable phases like fcc-(Ti,Al)N and fcc-(Al,Ti)N is facilitated by intermixing of Ti and Al during the physical vapour deposition process and by a limited mobility of the deposited species, which already hinders the decomposition of the metastable compounds during the deposition process. The adatom mobility increases with increasing substrate temperature and decreases with increasing bias voltage. Thus, these parameters of the deposition process are crucial for tailoring the thin-film microstructure.

A high adatom mobility, which was accomplished by the deposition temperature of 723 K and a low bias voltage (*U*_B_ = −40 V) in our samples, already leads to a relaxation of the residual stress during the deposition process. Furthermore, it cannot prevent the segregation of TiN and AlN in supersaturated Al-rich (Ti,Al)N. The locally Al-enriched (Al,Ti)N forms highly faulted wurtzitic clusters, which are detectable in the XRD patterns as ‘diffuse scattering’ or as very broad diffraction lines. Preferentially, the w-(Al,Ti)N clusters grow on fcc-(Ti,Al)N with the habitus planes 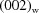 and 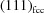. This mutual orientation relationship is facilitated by similar symmetry operations, which exist along the 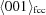 direction in the wurtzitic crystal structure and along the 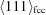 direction in the fcc crystal structure (Rafaja *et al.*, 2014 ▶). Still, owing to the different size of the fcc and wurtzitic elementary cell within the respective habitus plane, this intergrowth increases the anisotropy of the elastic constants determined from the dependence of the measured cubic lattice parameters on the diffraction indices as discussed above. The high adatom mobility promotes the formation of fcc-(Ti,Al)N crystallites having a size between 10 and 12 nm. Owing to the heteroepitaxy between fcc-(Ti,Al)N and w-(Al,Ti)N, and to the small size of the w-(Al,Ti)N domains, the segregation of elements and even the formation of w-(Al,Ti)N domains do not lead to an additional fragmentation of the fcc-(Ti,Al)N crystallites. However, these phenomena are still recognized by the broadening of XRD lines from fcc-(Ti,Al)N as increasing microstrain.

At the lower adatom mobility that was achieved *via* higher bias voltages, TiN and AlN segregate as well, but Al-rich (Al,Ti)N does not form visible wurtzitic clusters in the composition range under study. An explanation of this effect is that the low adatom mobility does not support the relaxation of the residual stress. The high compressive residual stress (8–14 GPa) present in the thin films deposited at *U*_B_ = −80 and −120 V stabilizes high-pressure fcc-(Al,Ti)N. Furthermore, the local accumulations of AlN result in a fragmentation of the fcc-(Ti,Al)N crystallites into nanocrystallites having a size between 3 and 4 nm. Adjacent fcc-(Ti,Al)N nanocrystallites are slightly misoriented, which was concluded from the loss of their partial coherence for X-rays.

## 4. Conclusions   

On the example of (Ti,Al)N thin films with a high aluminium content, it was illustrated that X-ray diffraction is a very efficient experimental method for the microstructure assessment of thermodynamically metastable compounds, for the description of their micromechanical properties and for the identification of the decomposition pathways. Detailed analysis of X-ray diffraction patterns yields information about the phase composition, lattice parameters, residual stress, anisotropy of the elastic constants, size of coherently diffracting domains and amount of the microstructure defects. In the supersaturated solid solution of titanium nitride and aluminium nitride, (Ti,Al)N, these microstructure features were employed to describe the microstructure formation as a function of the surface mobility of the deposited atoms for different high degrees of the thermodynamic instability. It was confirmed that a higher degree of thermodynamic instability forces the segregation of TiN and AlN and that the surface mobility of deposited atoms affects the decomposition mechanism and the distribution of the segregated species already in the deposition process. A high atomic mobility (achieved by a low bias voltage during the cathodic arc evaporation process) was shown to contribute to the relaxation of the intrinsic residual stress in fcc-(Ti,Al)N and thus to the formation of wurtzitic AlN during segregation of the excessive AlN from (Ti,Al)N. The wurtzitic AlN was located predominantly outside of the fcc-(Ti,Al)N domains, which consequently persisted unfragmented. Some AlN was accommodated inside of the fcc-(Ti,Al)N domains, where it increased the shear stiffness. Low atomic mobility (accomplished by high bias voltage in the deposition process) was found to promote the formation of high compressive residual stresses, which stabilize the high-pressure fcc-AlN. Part of the aluminium-rich fcc-(Al,Ti)N starts to transform into w-(Al,Ti)N *via* shearing, which is manifested by a decrease of the shear stiffness of the cubic structure. The excessive AlN segregates from fcc-(Ti,Al)N, but it is located inside of the fcc-(Ti,Al)N domains, where it leads to their fragmentation and to the formation of nanosized fcc-(Ti,Al)N crystallites.

## Figures and Tables

**Figure 11:**
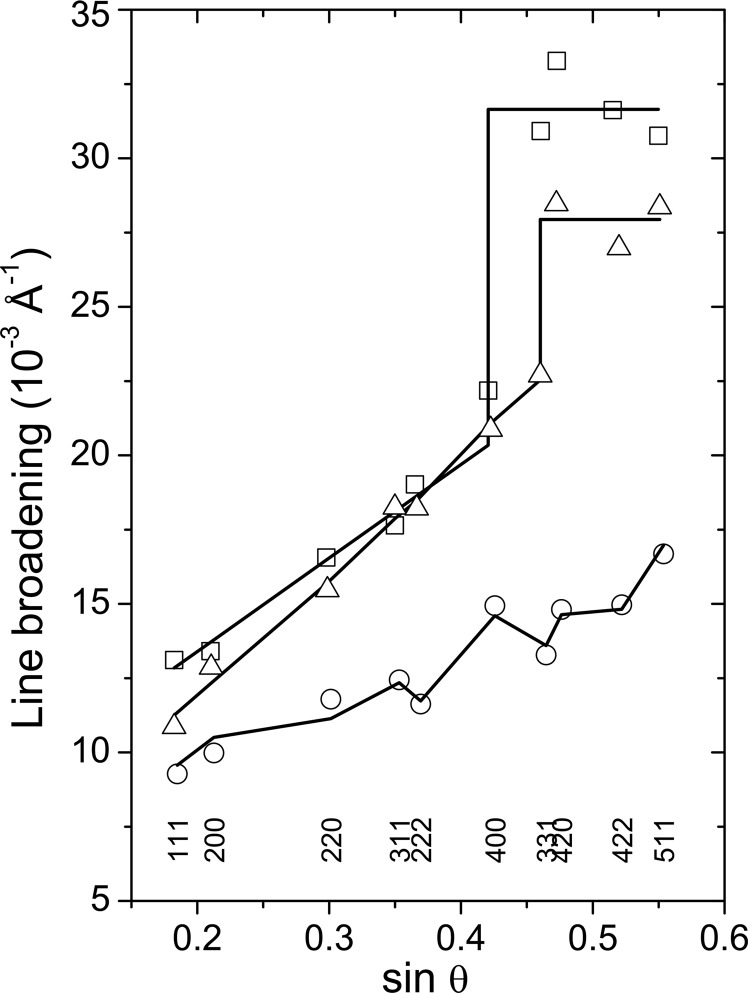
Diffraction line broadening measured for fcc-(Ti,Al)N in Ti_0.44_Al_0.56_N and plotted as a function of 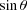. Circles denote the line broadening measured for *U*_B_ = −40 V, boxes for *U*_B_ = −80 V and triangles for *U*_B_ = −120 V. The approximation of the line broadening was performed analogously to Fig. 10 ▶.
